# Supramolecular Protein Assemblies: Building Blocks, Organism- or Cell-Specific Varieties, and Significance

**DOI:** 10.3390/biom14111342

**Published:** 2024-10-22

**Authors:** Victoria I. Bunik

**Affiliations:** 1 Belozersky Institute of Physicochemical Biology, Lomonosov Moscow State University, 119991 Moscow, Russia; bunik@belozersky.msu.ru; 2 Faculty of Bioengineering and Bioinformatics, Lomonosov Moscow State University, 119991 Moscow, Russia; 3 Department of Biochemistry, Sechenov Medical University, 105043 Moscow, Russia

In recent decades, biology has made tremendous progress in the high-throughput analytic and genetic techniques used to characterize the molecular components of living cells and their interactions. The structural characterization of proteins has developed from the X-ray and/or NMR resolution of smaller proteins or protein domains to the cryoelectron microscopy (cryo-EM)-assisted understanding of the organization of the macromolecular ensembles comprising these smaller parts. The associated terminology has developed accordingly, with such terms as the protein tertiary and quaternary structure, protein complexes, and multienzyme complexes increasingly being substituted by supercomplexes, macromolecular or supramolecular ensembles/assemblies/structures, and molecular machines. At the same time, with the progress in metabolomics, the usage of the term “metabolon”, describing the supramolecular organization of the consecutive enzymes of the same pathway [1,2,3] and popular in the 1970s, has been disappearing—probably to avoid confusion with the metabolomics term “metabolome”. Unfortunately, the choice of term often depends on the scientific school to which the research belongs rather than specific meaning of the term, and is also affected by personal preference regarding the use of metaphors like “molecular machines”. To more easily understand what we are talking about, stronger definitions in the field of the supramolecular organization of proteins are desired. This has manifested in some attempts to establish such definitions, mostly deriving from the field of chemistry [4,5]. The term “protein molecule” is the most ambiguous one. Depending on the context, it may refer to a protein monomer or to a protein supramolecular structure. However, protein monomers may oligomerize to different degrees, forming complexes with other proteins. Furthermore, functional proteins may have a different supramolecular organization even within one organism, e.g., dependent on the tissue [6]. Regarding a protein, not only may its oligomerization degree or oligomerization type (homo or hetero) vary, but the polypeptide chain may undergo multiple post-translational modifications. In contrast, the composition of a molecule, as well as its structure-defined properties, are assumed to be constant. Hence, the term “protein molecule” is conditional, and may not be used without specifications regarding the considered molecule composition. In this Special Issue dedicated to protein supramolecular structures, we would like to promote the terms which have more specified meanings. Terms such as protein monomer, dimer, tetramer, homo- or heterooligomer are not only more defined than the term “protein molecule”, but also stress the possibility of multiple compositions and oligomeric states of a protein, underlying its different functions or regulation. The protein molecule is most specifically defined by its gene-encoded amino acid sequence.

The papers collected in this Special Issue exemplify the different levels of the protein supramolecular assemblies and their building blocks, covering the recent developments in deciphering the structural determinants and functional significance of protein oligomerization.

## 1. Homo- and Heterooligomerization of a Protein as the Building Elements of a Supramolocular Level of Protein Structures

A folded polypeptide chain of a protein represents the protein monomer with its specific tertiary structure. Functional proteins are rarely monomeric. The quaternary structure of a protein refers to the oligomerization of the protein monomers, which in this case may also be called “subunits”, implying the subordination of such monomers within an oligomer. A quaternary level of structural organization is common for mature functional proteins. The oligomerization of a single type of subunits results in homooligomers, exemplified by the tetrameric structure of the central enzyme of glycolysis, glyceraldehyde-3-phosphate dehydrogenase (GAPDH), considered in the paper of Muronetz et al. [7], or the dodecameric structure of archeal ketol–acid reductoisomerase, which is important for the biosynthesis of branched-chain amino acids, as presented by Lemair et al. [8]. The oligomerization of different types of protein monomers (or subunits) results in heterooligomers. The review of Qin et al. [9] provides many examples of the very complex heteromeric structures inherent in mitochondrial membrane proteins, such as F_1_F_o_-ATP synthase or respiratory complexes. In some cases, such heterooligomers behave as monomer-like entities, undergoing further “dimerization”, as exemplified by the cytochrome *b6/f* complex. In this complex, two heterooligomers, each comprising eight to nine polypeptide subunits, interact with the formation of the dimer-like structure of 220 kDa [9]. This property of a heterooligmer, which can further oligomerize with the same oligomeric unit, as if they were monomers, may be better described by using the term “protomer” for the oligomerization unit. A much simpler example to clarify the definition of protomer is the stable association of the α and β subunits within heterotetrameric mammalian pyruvate dehydrogenase, comprising the two heterodimeric protomers. These protomers are analogous to the two monomers, each formed by a single polypeptide chain, within the homodimer of bacterial pyruvate dehydrogenases [10].

Oligomerization may be regulated by different protein conformations, which may be stabilized by post-translational modifications or substitutions in the amino acid residues of a polypeptide chain. For instance, oxidative modifications to a reactive cysteine residue of GAPDH lead to the dissociation of the tetramer [7]. Post-translational modifications greatly affect the heterologous complexes of transcriptional regulator p53, as reviewed by Zavileyskiy and Bunik [11]. In other cases, the substitution of Tyr to Cys due to the editing of mRNA by adenosine desaminase increases the protein’s propensity to amyloidogenic transformation [12]. GroEL-like chaperonins of bacteriophages catalyze the ATP-dependent conversion of prion protein monomers into short amyloid fibrils, which further aggregate into large clusters [13]. A similar stimulation of the fibrillar transformation occurs with synuclein [14]. Intrinsically unstructured regions of a protein become structured within its heteromeric complexes, emphasizing the role of conformational changes in the protein during protein–protein interactions [11]. Partial proteolysis or other conditions that occur during the isolation of supramolecular protein structures, which lead to their dissociation into the protein components, may interfere with the characterization of biologically relevant supramolecular structures and their catalytic activities. This is exemplified by a high-molecular-weight system for the biosynthesis of an alarmone, such as adenosine thiamine triphosphate, as described in the review of Bettendorf [15].

## 2. Supramolecular Assemblies of Multiple Proteins

The basic types of the protein supramolecular structures described above, i.e., homo- and heterooligomers, are employed in a combinatorial way to create a higher level of complexity, which is required to build supramolecular or macromolecular assemblies/ensembles, also called “molecular machines”. While “molecular machine” is a metaphor, “macromolecular/supramolecular assembly, ensemble or structure” pinpoints such essential meanings as “interaction” and “macromolecule”. The term “supramolecular” is preferred here, as it better stresses the subordination of structural levels, defined by the interactions between macromolecules. Besides, the term does not exclude the role of small molecules, such as lipids, in the formation of supramolecular assemblies. The usage of the terms “assembly” and “structure” may accentuate the functional/assembling and structural aspects, correspondingly.

An essential feature of supramolecular assemblies is their reliance on the non-covalent interactions between their multiple proteins and other components. Each of the proteins in the assembly is represented by the folded protein polypeptide (subunit, or monomer), individually encoded by a separate gene, with the protein monomers or protomers further oligomerizing into the more complex structures. The major types of supramolecular assemblies are as follows:

- Multienzyme complexes and supercomplexes, which employ the non-covalent binding of enzymes and/or regulatory proteins with different activities to increase the efficiency of catalysis and/or regulation, as exemplified in [9,11,15];
- Supramolecular assemblies created for energy storage in the form of an “energized” protein conformation, which may then be relaxed for an energy-requiring process, such as chemical synthesis, membrane transport, etc. [9];
- Supramolecular assemblies involving not only proteins, but also nucleic acids, as exemplified by ribosomes and nucleosomes;
- Biological polymers, such as microtubules or viral envelop proteins. In contrast to oligomerization, which implies hundreds of monomeric/protomeric protein units, polymeric structures involve thousands of such units.

Below, we consider the first two types of supramolecular assembly considered in the papers collected in our Special Issue in more detail.

### 2.1. Multienzyme Complexes

Along with the accumulated knowledge of the structure, function, and regulation of “classic” multienzyme complexes, such as those of 2-oxo acid dehydrogenases [10] or fungal fatty acid synthetases [16], there has been a growing understanding that new biological properties arise in the supramolecular structures. Multienzyme complexes demonstrate how the building blocks of the protein supramolecular structures, i.e., the homo- and heterooligomers with a low oligomerization degree, further organize into assemblies with a higher oligomerization degree and increased heterogeneity, and the advantages of this oligomerization. As the mechanisms of formation and regulation of protein complexes are similar for proteins with enzymatic, regulatory, and/or scaffolding functions, studies of classic multienzyme complexes have greatly promoted research on protein supramolecular organization in general. A brief overview of this knowledge, provided here, covers the homooligomeric proteins and supercomplexes considered in our Special Issue.

Multienzyme complexes of 2-oxo acid dehydrogenases [10] or fungal fatty acid synthetases [16] have long been a paradigm in scientific research aiming to understand the structural determinants and biological role of protein supramolecular organization. Each enzyme of the complex may form binary, ternary, etc., protein complexes, employing both homo- and heterooligomerization. Moreover, dependent on the biological system, the active site coupling in these multienzyme complexes may be further increased by evolution. That is, the separate enzymatic components that form the complex through non-covalent interactions, may be substituted with multifunctional enzymes. Multifunctional enzymes have different active sites with different catalytic activities within one polypeptide chain encoded by one gene. The active sites of the multienzyme complex components may be merged within one polypeptide, either partly or in full. For instance, in fungi, fatty acid synthetase is a 2.6 MDa heterododecamer α_6_β_6_, comprising six copies of each of the two different polypeptides. Each polypeptide possesses four different active sites which are covalently linked within the polypeptide structure. The combination of two polypeptides, each containing different active sites, within the multienzyme complex is supported by the non-covalent binding between the α and β subunits. This non-covalent complex can catalyze all eight different reactions of the fungal fatty acid synthesis [17,18]. In contrast, the mammalian fatty acid synthetase is a homodimer, with each of its monomers representing multifunctional enzymes comprising all the active sites for mammalian fatty acid synthesis [19,20].

Multienzyme complexes of 2-oxo acid dehydrogenases may merge their active sites, too. For example, the first and second catalytic components merge in the 2-oxoglutarate dehydrogenase complex of *Mycobacteria* and *Actinobacteria* [21,22]. This entails changes in the overall supramolecular structures of multienzyme complexes, that depends on the component polypeptides. The cubic 24-meric core of the second component of 2-oxoglutarate dehydrogenase complex is evolutionary conserved in the organisms with the three enzymatic components, encoded by the component-specific genes [23]. When the catalytic activities of the first and second enzymatic components of the 2-oxoglutarate dehydrogenase complex merge within one polypeptide, encoded by a single gene, the complex is organized around the trimer formed by the acyl-transferase domain of the hybrid enzyme component [21]. This type of organization of the 2-oxoglutarate dehydrogenase complex is associated with the organism-specific regulation of the overall reaction by a protein inhibitor, and with the specific intracellular localization of the complex [22,23].

Thus, multienzyme complexes are built from the enzymes encoded by different genes to efficiently catalyze and regulate a block of consecutive chemical reactions. A specific feature of the “classic” multienzyme complexes, such as those considered above for multienzyme complexes of 2-oxo acid dehydrogenases or fungal fatty acid synthetases, compared to metabolones or supercomplexes, is that some of the reaction substrates or intermediates are covalently bound to the complex components. As a result, at least some enzymes of a classic multienzyme complex lack their complex-supported activities outside the complex. In contrast, the consecutive reactions catalyzed by the enzymes of a metabolic pathway within a metabolon or supercomplex, such as purinosome [9], are also catalyzed by the separate enzymes outside the complex. This feature of metabolones or supercomplexes may be employed to use the branch-point enzymes of these supramolecular structures in different pathways. The complexes between different enzymes may form not only to increase the catalytic efficiency, but also to regulate substrate fluxes. For instance, the complex between GAPDH and phosphoribulokinase, where the catalytic activities of the enzymes are inhibited, is formed for regulatory purposes [9].

### 2.2. Supercomplexes

As discussed above, the term “supercomplex” often substitutes the more specific term “metabolon”. In such cases, the complexes of enzymes (with each having its own supramolecular structure) catalyze a chain of consecutive reactions along a metabolic pathway, such as glycolysis [3], the respiratory chain, or purine synthesis [9]. That said, supercomplexes are not limited to metabolones, as they may also involve interactions between the distant enzymes of the pathway [24] or they may be formed for regulatory purposes, such as the supercomplex between GAPDH and phosphoribulokinase [9]. Similar to multienzyme complexes, the interactions supporting supercomplexes are non-covalent. However, in contrast to multienzyme complexes, the formation of supercomplexes is not obligatory for each of the separate catalyzed reactions to occur. The conditions may require the enzymes in the branched points of metabolism, i.e., the enzymes participating in different pathways, to change their abundance in the supercomplexes of these different pathways. Accordingly, the interactions in supercomplexes are transient and depend on the specific metabolic conditions. For instance, the assembly of purinosomes from their separate enzymatic components is induced under an increased demand for purines [9]. Using cryo-EM, the structures of the plant and mammalian supercomplexes of the respiratory chain (also called respirasomes) were characterized [9,25]. Cryo-EM images of the proteolipid structure of cristae in the mammalian cardiac mitochondria show parallel rows of respirasomes and dimerized heterooligomeric protomers of F_1_F_o_-ATP synthase [25]. The resulting architecture is called an “ordered cluster” by the authors. In some regions of the cristae, the respirasome complexes I or IV are docked to F_1_F_o_-ATP synthases, while in other regions, the distance between the complexes is longer. The dynamic nature of such interactions may affect the cristae morphology [26].

### 2.3. Supramolecular Protein Assemblies for the Energy Transformation

A paradigm of the energy-transforming protein system is F_1_F_o_-ATP synthase. This membrane-bound supramolecular assembly demonstrates a very complex heterooligomeric organization to synthesize the universal energy equivalent of biological systems, ATP, from ADP and inorganic phosphate (P_i_). The synthesis uses the energy of the proton gradient through the inner mitochondrial membrane, the so-called proton-motive force (pmf). The recently characterized structure of human F_1_F_o_-ATP synthase [27] is principally similar to that of the plant system [9], although there are specific regulatory differences that make the plant system light-dependent. These differences may be used to solve specific challenges. For instance, a comparative structure–function analysis of human and pathogen F_1_F_o_-ATP synthases is ongoing, with the aim of using this system for the development of antibiotics [28]. The currently available structures of F_1_F_o_-ATP synthases take into account a tremendous amount of previous long-lasting studies on the function, composition, and structure of this system. In particular, the enzyme of bovine mitochondria has long been studied as a surrogate of the human F_1_F_o_-ATP synthase [29]. Including the transiently associated inhibitory protein, the heterooligomeric protomer of F_1_F_o_-ATP synthase, which performs ATP synthesis, is assembled from 29 subunits of 18 different proteins [30]. In mitochondrial studies, the association of two or four such protomers has been observed, referred to as dimers or tetramers of “monomeric” F_1_F_o_-ATP synthase [29]. Using the term “monomer” for a highly heterogeneous oligomer without providing any specifications regarding the implied heterooligomeric composition is rather misleading. Regarding F_1_F_o_-ATP synthase, the term “protomer”, implying the heterooligomerization of the structural unit, would be much more appropriate than “monomer”. The ambiguity in the definitions of monomer and dimer for the F_1_F_o_-ATP synthase is further highlighted by the fact that different combinations of the protein subunits of F_1_F_o_-ATP synthases are characterized in different studies. Furthermore, the symmetry of the structural organization of the catalytic subunits of the F_1_ head (heterohexamer composed of three αβ protomers) and membrane-embedded proton-translocating cylinder of F_o_ (homooctamer) is principally different. Finally, the dimerization of the F_1_F_o_-ATP synthase strongly differs from the usual monomeric dimerization in the interface formation. The symmetrically organized heterooligomer subunits of F_1_F_o_-ATP synthase do not participate directly in the interface combining the two protomers. Therefore, this particular type of dimerization of supramolecular structures would be more appropriately referred to as the dimerization of specific parts/subunits of the F_1_F_o_-ATP synthase heterooligomer. Remarkably, only the non-symmetrical part of the protomers, i.e., the regulatory membrane subunits, interacting with the F_o_ cylinder, participate in the dimerization of the F_1_F_o_-ATP synthase protomers [29]. The ensuing symmetry of the F_1_F_o_-ATP synthase “dimer” emphasizes the role of symmetry in the self-organization of supramolecular assemblies. 

Another misleading piece of terminology in the field of the F_1_F_o_-ATP synthase research concerns the application of the term “domain” to the hexameric F_1_ head or to the octameric F_o_ proton channel [31]. At least when the enzyme structure is discussed, the term “domain” should be employed as a term of structural biology, implying a part of the polypeptide that is able to acquire a tertiary structure upon folding.

In vivo, the aforementioned protein composition of F_1_F_o_-ATP synthase includes also the phospholipids that fill in the gaps between the two interacting protomers [29], and proteins of the mitochondrial cristae organization complex [26]. Although structural descriptions of assemblies such as F_1_F_o_-ATP synthase are not straightforward, more accurate and unified terminology is definitely required to promote understanding throughout the protein research field, involving combined efforts from biologists, chemists, and physicists. In particular, the terms “protomer” or “heterooligomer” for F_1_F_o_-ATP synthase are much more appropriate than “monomer”. Regarding the “dimerization of F_1_F_o_-ATP synthase”, an unambiguous indication that the dimerization involves the membrane-embedded regulatory subunits would definitely be more concise and hence more informative. Using a correct and unified terminology is especially important when systems less known than F_1_F_o_-ATP synthase, or unusual functions of F_1_F_o_-ATP synthase, are considered.

Intriguingly, F_1_F_o_-ATP synthase may use a proton gradient across the mitochondrial membrane to synthesize not only ATP from ADP and P_i_, but also a structurally similar thiamine triphosphate, a potential alarmone and/or metabolic regulator, from the essential coenzyme of glucose oxidation, thiamine diphosphate, and P_i_ [15]. This function of F_1_F_o_-ATP synthase is observed in the brain, but not in the liver. The available data suggest that the thiamine triphosphate synthesis depends on the brain-specific generation of a yet-unknown regulator of F_1_F_o_-ATP synthase [15].

As mentioned above, deciphering the composition and structure of such a complex heterooligomer as F_1_F_o_-ATP synthase has required the long-lasting efforts of many researchers worldwide. The isolation of supramolecular assemblies of this sort is challenging, as non-covalent interactions are easily disrupted during the isolation, leading to the loss of their biologically relevant catalytic activities and/or regulatory properties. For instance, in contrast to the complete structure of the membrane-bound heterooligomer synthesizing ATP, the solubilized F_1_ part of F_1_F_o_-ATP synthase catalyzes only the opposite reaction of ATP hydrolysis. The easy dissociation of the non-covalent protein complexes upon isolation hinders the characterization of supramolecular assemblies. For instance, it is known that a large supramolecular assembly is involved in the synthesis of adenosine thiamine triphosphate, a metabolic regulator which accumulates in *Escherichia coli* in response to carbon starvation. However, this activity is lost upon isolation, and the enzyme(s) synthesizing the derivatives have not been identified to date [15].

## 3. Advantages of Protein Supramolecular Assemblies

### 3.1. Regulatory Significance

The interacting protein interfaces in an oligomer enable cooperative action among the subunits, usually quantified by Hill’s coefficient. This cooperativity allows the protein oligomer to be much more sensitive to changes in the surrounding medium, e.g., changes in the concentration of the protein ligand, than the non-cooperative system. In a monomer, the cooperative effects require an additional ligand-binding site. In an oligomer, the monomeric units, each containing the only ligand binding site, allow the multiple binding sites of the oligomeric structure to cooperate. The higher the number of interacting monomers, or protomers, in an oligomer, the higher their cooperativity. The highly regulated homooligomers are exemplified by a tetramer of GAPDH [7], or a dodecamer of ketol–acid reductoisomerase [8]. The cooperative formation of the active dodecamer of glutamine synthase in an archebacterium is induced by an allosteric regulator of the process, 2-oxoglutarate, through its binding at the interface between the two monomers of the glutamine synthase [32]. This mechanism allows for the biologically relevant regulation of the nitrogen assimilation. The regulatory significance of homooligomerization is further supported by the existence of different oligomeric forms of the same enzyme, either within one organism [6,7,32] or in different organisms [8,10].

The regulatory significance of heterooligomerization is demonstrated by the evolvement of mammalian heterotetrameric pyruvate dehydrogenase, as discussed above, or a 60-meric core of the mammalian pyruvate dehydrogenase complex, comprising the catalytic component acetyl dihydrolipoamide transferase and its isoenzyme, the so-called protein X, whose function is not the catalysis but the binding of the terminal catalytic component [10]. The regulatory, rather than catalytic, role of these heterooligomerizations is supported by the existence of homooligomeric pyruvate dehydrogenase and its multienzyme complex core in bacteria. Splitting a single polypeptide chain of a monomer into the α and β subunits of a protomer adds regulatory opportunities at the transcriptional level, as each subunit is encoded by its own gene. Besides, segregation of the two functions, such as catalysis and component binding, within the functions-specialized subunits forming the pyruvate dehydrogenase complex core, creates additional mechanisms to regulate the terminal enzyme component stoichiometry in the mammalian complex, compared to its bacterial counterpart.

The formation of supercomplexes may also lead to “molecular symbiosis”, creating new regulatory properties during the interaction between the supercomplex enzymes. For instance, in *Mycobacterium tuberculosis*, a new allosteric site is created in the supercomplex of the enzyme, starting the biosynthesis of aromatic amino acids, and chorismite mutase, controlling the branch point of the pathway [24].

Different oligomeric forms of a protein may have different functions and/or regulation mechanisms. Therefore, they are often observed in the highly regulated metabolically essential proteins or transcriptional regulators. For instance, the dissociation of a catalytically active tetramer of GAPDH into monomers may be induced by oxidative modifications, resulting in nuclear localization and the moonlighting function of the enzyme in an apoptotic cascade [7]. Furthermore, transcriptional regulation requires tetramers of p53, while the apoptosis-relevant interaction with Bcl and Bax proteins is supposed to involve the p53 dimers [11].

The heterooligomerization of proteins is a key component of signal transduction. This is demonstrated by a high number of heterologous complexes inherent in master regulators, such as the transcriptional regulator of energy metabolism p53 [11] or mammalian target of rapamycin (mTOR), responding to nutrient cues and metabolic stress, including changes in aging [33,34,35,36]. The heterooligomerization of regulatory proteins with different partner proteins is supported by intrinsically disordered regions in the regulatory proteins. These highly flexible regions represented by conformational ensembles [37], acquire their structure in the heterologous complexes, exemplified by structural changes in the heterologous complexes of p53 [11], or upon the binding of 2-oxoglutarate dehydrogenase to other components of the multienzyme complex, mediated by the unstructured N-terminal region of 2-oxoglutarate dehydrogenase [38,39,40].

### 3.2. Catalytic Advantages

The formation of the enzyme oligomers is usually significant for the catalytic or binding function, even when each subunit of a homooligomeric enzyme possesses all the residues required for catalysis. In many cases, only the spatial organization of dimers or trimers enables catalysis, as the amino acid residues of functionally competent active sites (two in a dimer, or three in a trimer) are contributed by different subunits. It may be hypothesized that this type of structural organization in enzymes evolved to more effectively employ the regulatory advantages of the interacting subunits and their active sites.

The supramolecular structure of the multienzyme complexes and supercomplexes often provides spatially separated “chambers”, increasing the local concentration of the reaction/pathway substrates and their intermediates. Direct tunneling within the multienzyme complexes is employed for intermediates that are toxic and/or entering an undesirable equilibrium, such as ammonia tunnels in ferredoxin-dependent glutamate synthetases [41].

## 4. Conclusions and Future Directions

The increased availability of the structural characterization of large protein assemblies by cryo-EM greatly adds to the knowledge gained from reductionistic studies of the isolated components of the protein supramolecular assemblies. Understanding the structure and function of supramolecular assemblies provides a scaffold that can be used to combine the information from deciphered genomes, tools for their manipulation, the resolved structures of varied proteins, and the functional parameters of proteins. Using all the accumulated knowledge to achieve a holistic understanding of biosystems will increase our ability to direct their regulation. Thus, knowledge of the structural interactions between particular cellular components and the functional consequences of the formation of supramolecular assemblies is greatly required. Understanding the principles and regulation of the homo- and hetero-oligomerization of proteins in vitro and in vivo, and of the role of these processes in the formation of cellular supramolecular assemblies, paves the way to acknowledging the role of cellular compartmentation in the sustainability of the living systems.
